# Long-Term Outcomes of Systemic Lupus Erythematous Patients after Pregnancy: A Nationwide Population-Based Cohort Study

**DOI:** 10.1371/journal.pone.0167946

**Published:** 2016-12-19

**Authors:** Ting-Fang Chiu, Ya-Wen Chuang, Cheng-Li Lin, Tung-Min Yu, Mu-Chi Chung, Chi-Yuan Li, Chi-Jung Chung, Wen-Chao Ho

**Affiliations:** 1 Department of Pediatrics, Taipei City Hospital, Zhongxiao Branch, Taipei, Taiwan; 2 Division of Nephrology, Taichung Veterans General Hospital, Taichung, Taiwan; 3 Management Office for Health Data, China Medical University Hospital, Taichung, Taiwan; 4 Department of Medicine, College of Medicine, China Medical University and Hospital, Taichung, Taiwan; 5 Department of Anesthesiology, China Medical University Hospital, Taichung, Taiwan; 6 Department of Health Risk Management, College of Public Health, China Medical University, Taichung, Taiwan; 7 Department of Medical Research, China Medical University Hospital, Taichung, Taiwan; 8 Department of Public Health, College of Public Health, China Medical University, Taichung, Taiwan; Kaohsiung Medical University Hospital, TAIWAN

## Abstract

**Background:**

Data on long-term maternal outcomes in patients with systemic lupus erythematosus (SLE) are lacking. The study aimed to explore the relationships among SLE, pregnancy, outcomes of end-stage renal disease (ESRD), and overall mortality.

**Methods:**

We established a retrospective cohort study consisting of four cohorts: pregnant (case cohort) and nonpregnant SLE patients, as well as pregnant and nonpregnant non-SLE patients. One case cohort and three comparison cohorts were matched by age at first pregnancy and index date of pregnancy by using the Taiwan National Health Insurance Research Dataset. All study subjects were selected based on the index date to the occurrence of ESRD or overall death. Cox proportional hazard regression models and Kaplan–Meier curves were used in the analysis.

**Results:**

SLE pregnant patients exhibited significantly increased risk of ESRD after adjusting for other important confounders, including immunosuppressant and parity (HR = 3.19, 95% CI: 1.35–7.52 for pregnant non-SLE; and HR = 2.77, 95% CI: 1.24–6.15 for nonpregnant non-SLE patients). No significant differences in ESRD incidence were observed in pregnant and nonpregnant SLE patients. Pregnant SLE patients exhibited better clinical condition at the baseline and a significantly lower risk of overall mortality than nonpregnant SLE patients.

**Conclusions:**

Our data support current recommendations for SLE patients to avoid pregnancy until disease activity is quiescent. Multicenter recruitment and clinical information can be used to further examine the association of SLE and ESRD (or mortality) after pregnancy.

## Introduction

Systemic lupus erythematosus (SLE) is a prototypical autoimmune disease that involves multiple organs, particularly the kidneys; SLE mostly affects female patients at child-bearing age [1]. A growing body of evidence indicated that pregnant SLE women are at a high risk of developing maternal and fetal complications [2]. Significant alternations in hormonal and immune activity in pregnant SLE patients may be attributed for these unwanted events [1, 3]. For example, many perinatal complications frequently develop in childbearing SLE patients, such as disease flare-up, a new onset of lupus nephritis (LN), and preeclampsia, which are associated with a higher possibility of undesired fetal outcomes, such as demise or intrauterine growth restriction. Studies verify that the flare-up rate in LN was as high as 25.6% (17.4%–33.8%) and increased the risk of maternal and neonatal morbidity [4]. Some hypertension-related diseases, such as pre-eclampsia or eclampsia, resulted in an incidence rate ranging from 11.0% to 35.0% in pregnant SLE women, which was approximately six-fold higher than that in the general population [1, 5, 6].

Aside from the uncommonly high immunological risk in pregnant SLE women, pregnancies inevitably cause difficulties to the kidneys during the gestational course [7]. As the kidneys undergo the gestational process, a corresponding physiological compensation, such as glomerular hypertrophy and hyper-filtration, would occur to meet the excessive requirements of pregnancy, which is a consequence of increased renal blood flow [7, 8]. In healthy women, the changes in the kidneys as a result of pregnancy would stabilize naturally without any sequel after the delivery of a child [7–9]. However, patients with chronic kidney disease (CKD) would probably fail to overcome these problems during pregnancy. These patients could more likely suffer permanent damage to the kidneys resulting in CKD because of the changes in chronic fibrosis caused by glomerular hyper-filtration. Data suggest that insufficient adaption of the kidneys was associated with the risk of chronic kidney failure and progression to end-stage renal disease (ESRD) (including dialysis and renal transplantation) after delivery [10, 11].

Up to 75% of pregnant SLE patients with or without abnormal serum creatinine changes exhibit a spectrum of various abnormal renal diseases [12]. Increased risk of activity flare-up in these patients would contribute to short-and long-term adverse effects on the kidneys, which could potentially lead to accelerated progression to ESRD [13, 14]. Recent data show dramatic improvements in live birth rate [2, 15, 16]; however, data about the long-term maternal outcomes after pregnancy are lacking. The major limitations of these studies were the low numbers of patients in a cross-sectional study design. This study aims to explore the association between pregnant SLE patients and renal outcomes by analyzing a nationwide database.

## Methods

### Data sources

Taiwan’s National Insurance Research Database (NHIRD) provided the data. The National Health Insurance (NHI) program in Taiwan is a compulsory universal health insurance program, which was established in 1995. NHIRD consists of health care data from more than 99% of the entire population of 23.74 million and includes comprehensive health care information. SLE and ESRD diagnoses were determined using the Registry for Catastrophic Illness Patient Database (RCIPD), which is a separate subpart of NHIRD. Histological or cytological evidence of the disease is required for patients before they are given a catastrophic illness certificate for SLE or ESRD and coded with International Classification of Diseases, Ninth Revision, Clinical Modification (ICD-9-CM). This study was approved by the Ethics Review Board of China Medical University (CMU-REC-101-012). The identifications, health-related records, and information of all insurant were de-identified and scrambled. International Classification of Diseases, Ninth Revision coding was used to classify the diseases in the study.

### Study subjects

A retrospective cohort study was established. SLE patients (ICD-9-code 710.0) with first pregnancy were identified as the case cohort (ICD-9 procedure 72–74 or ICD-9 code 640.x1-676.x1, 640.x2-676.x2, 650–659) in the database from January 1997 to December 2010. The date of first pregnancy in pregnant SLE patients was defined as index date. Three comparison cohorts based on the index date of case cohort or/and incident date of SLE were constructed as follows: comparison of Cohort 1, SLE patients without any pregnancy during 1997–2010 and matched by the incident date of the SLE of the case cohort; comparison of Cohort 2, non-SLE women with the same index date of the case cohort; and comparison of Cohort 3, non-SLE women without any pregnancy during 1997–2010. All the participants with a baseline history of ESRD (ICD-9 code 585) before the index date were excluded. Three comparison cohorts was approximately 1:2 matched by age at first birth of the case cohort (every five years).

### Outcome measurement and comorbidities

The main endpoints of study were the incidences of ESRD or death from 1997 to 2010. All of the study subjects were selected based in the index date to the occurrence of ESRD, death, loss to follow-up, withdrawal from the database, or the end of 2010, which comes first. ESRD included patients receiving hemodialysis, peritoneal dialysis, and renal transplantation. Inpatient diagnosis records were incorporated to ascertain the baseline comorbidities, including diabetes mellitus (ICD-9 code 250) and hypertension (ICD-9 code 401–405), CKD (ICD-9 code 585), proteinuria (ICD-9 code 791.0), pre-eclampsia (ICD-9 code 642.4x, 642.5x and 642.7x), eclampsia (ICD-9 code 642.6x), anti-phospholipid syndrome (APS) (ICD-9 code 795.79), Sjogren’s syndrome (SS) (ICD-9 code 710.2), infection (included urinary tract infections ICD-9 code 599.0, acute pyelonephritis ICD-9 code 590.10, pneumonia and influenza ICD-9 code 480.0–487.8, septicemia ICD-9 code 038.0–038.9), and stroke (ICD-9 code 430, 431, 432.0, 432.1, 432.9, 433.01, 433.11, 433.21, 433.31, 433.81, 433.91, 434.01, 434.11, 434.91). The analysis also considered the types of pregnant women receiving prenatal care from institutions, immunosuppresant treatment, including prednisolone, Endoxan, and Imuran, and parity of pregnancy.

### Statistical analysis

Data analysis first compared the distributions of age (≤ 20, 21–30, 31–40, > 40 years), residential areas (northern, central, southern, and eastern Taiwan), urbanization level, occupation (white collar, blue collar, and others), and baseline comorbidities between case cohort and individual comparison cohort. Chi-square test for categorical variables and t-test for continuous variables were used in the analysis. The incidence densities of ESRD and overall mortality were estimated in follow-up. The stratified results were compared by comorbidities. Multiple Cox proportional hazard regression models were utilized to estimate hazard ratios (HRs), the 95% confidence interval (CI) of the ESRD, and the overall mortality after adjusting sociodemographic factors and comorbidities. The event-free survival functions were assessed using the Kaplan-Meier method, and the differences were estimated by utilizing the log-rank test. All statistical analyses were performed using SAS statistical software (version 9.2 for Windows; SAS Institute, Inc., Cary, NC, USA). A two-tailed p value of <0.05 was considered statistically significant.

## Results

The mean age of all four cohorts was approximately 29 years (Table 1). Among the four cohorts, approximately half of the study participants were 21–30 years of age, resided in northern Taiwan, and had a white-collar jobs. The probability for pregnant SLE patients to have infection, stroke, CKD, proteinuria is lower than that of nonpregnant SLE patients. The prevalence of APS, SS, infection, stroke, CKD, and proteinuria (all p values < 0.05) in pregnant SLE patients was higher than that of non-SLE women who were pregnant or not.

**Table 1:** Demographic characteristics and comorbidities of the SLE patients and three comparison cohorts.

	SLE				p-value ^a^	Non-SLE					p-value ^c^
	Pregnant (N = 1526)		Non-pregnant (N = 2932)			Pregnant (N = 3052)		p-value ^b^	Non-pregnant (N = 3052)		
	n	%	n	%		n	%		N	%	
Age, yearMean (SD)	29.4	4.56	29.0	5.36		29.3	4.63		29.4	5.03	
≤20	33	2.16	65	2.22	0.72	66	2.16	0.99	66	2.16	0.99
21–30	818	53.6	1622	55.3		1636	53.6		1636	53.6	
31–40	659	43.2	1213	41.4		1318	43.2		1318	43.2	
41–50	16	1.05	32	1.09		32	1.05		32	1.05	
Geographic region					0.01			0.001			<0.001
Northern	714	46.8	1441	49.2		1529	50.1		1639	53.7	
Central	364	23.9	567	19.3		597	19.6		558	18.3	
Southern	356	23.3	731	24.9		685	22.4		674	22.1	
Eastern	92	6.03	193	6.58		241	7.90		181	5.93	
Urbanization					0.001			0.001			0.001
1 (highest)	449	29.4	1033	35.2		949	31.1		1015	33.3	
2	548	35.9	942	32.1		975	32.0		961	31.5	
3	245	16.1	463	15.8		549	18.0		571	18.7	
4 (lowest)	284	18.6	494	16.9		579	19.0		505	16.6	
Occupation					0.0003			0.04			<0.001
White collar	960	62.9	1662	56.7		1851	60.7		1549	50.8	
Blue collar	448	29.4	1004	34.2		897	29.4		1173	38.4	
Others	118	7.73	266	9.07		304	9.96		330	10.8	
Prenatal care institution								<0.001			
Medical center	878	57.5				479	15.7				
Regional hospital	232	15.2				682	22.4				
District hospital	262	17.2				995	32.6				
Clinic	154	10.1				896	29.4				
Comorbidity											
Diabetes	19	1.25	20	0.68	0.06	38	1.25	0.99	38	1.25	0.99
Hypertension	176	11.5	344	11.7	0.84	352	11.5	0.99	352	11.5	0.99
Mild or unspecified pre-eclampsia	94	6.16	1	0.03	<0.001	52	1.70	<0.001	0	0.00	-
Severe pre-eclampsia	39	2.56	0	0.00	-	54	1.77	0.08	0	0.00	-
Pre-eclampsia	8	0.52	0	0.00	-	24	0.79	-	0	0.00	-
Eclampsia	11	0.72	0	0.00	-	6	0.20	0.01	0	0.00	-
APS	74	4.85	165	5.63	0.27	2	0.07	<0.001	2	0.07	<0.001
SS	16	1.05	27	0.92	0.68	2	0.07	<0.001	2	0.07	<0.001
Infection	458	30.0	1039	35.4	0.0003	280	9.17	<0.001	227	7.44	<0.001
Stroke	42	2.75	124	4.23	0.01	27	0.88	<0.001	53	1.74	0.03
CKD	325	21.3	827	28.2	<0.001	7	0.23	<0.001	9	0.29	<0.001
Proteinuria	19	1.25	71	2.42	0.01	3	0.10	<0.001	2	0.07	<0.001
Immunosuppressant											
Prednisolone	1498	98.2	2877	98.1	0.92	92	3.01	<0.001	149	4.88	<0.001
Endoxan	501	32.8	1263	43.1	<0.001	17	0.56	<0.001	23	0.75	<0.001
Imuran	855	56.0	1947	66.4	<0.001	3	0.10	<0.001	5	0.16	<0.001
Parity								<0.001			
One	943	61.8				1635	53.6				
Two	480	31.5				1192	39.1				
More than three	103	6.75				225	7.37				

^a^ *p* values for comparison between SLE patients with and without pregnancy.

^b^ *p* values for comparison between pregnancy subjects with and without SLE.

^c^ *p* values for comparison between SLE patients with pregnancy and non-SLE without pregnancy.

**Fig 1:**
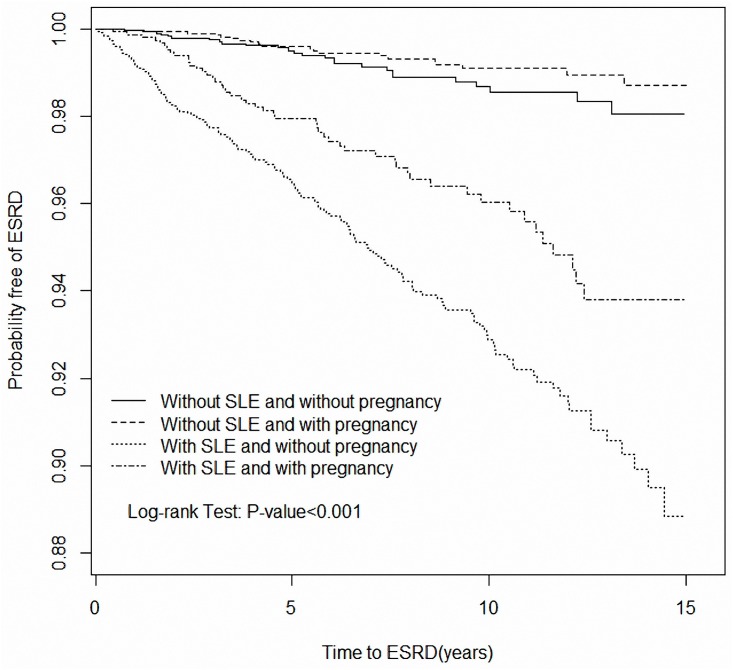
Survival curve of ESRD for SLE patients with and without pregnancy, and non-SLE women with pregnancy.

Fig 1 illustrates the different survival of ESRD among four cohorts (*p* < 0.001). Pregnant and nonpregnant SLE patients showed a similar outcome in terms of ESRD survival without statistical difference (*p* = 0.62) (data not shown). However, pregnant and nonpregnant SLE patients exhibited significantly higher incidences of ESRD than non-SLE women who were pregnant or not. After adjusting for potential confounders, pregnant SLE patients were associated with a statistically significantly higher risk of ESRD than non-SLE women who were pregnant (adjusted HR = 3.19, 95% CI = 1.35–7.52), particularly for patients with hypertension (Table 2). Similar results were observed when pregnant SLE patients were compared with non-SLE who were not pregnant (adjusted HR = 2.77, 95% CI = 1.24–6.15).

**Table 2:** Incidence rate and hazard ratios of ESRD stratified by different study variables.

	SLE				Adjusted HR (95% CI)^a^	Non-SLE		Adjusted HR (95% CI)^b^	Non-SLE		Adjusted HR (95% CI)^c^
	Pregnancy		Non-Pregnancy			Pregnancy			Non-Pregnancy		
	Case	Rate^#^	Case	Rate^#^		Case	Rate^#^		Case	Rate^#^	
Total	50	4.27	161	7.52	0.81 (0.57, 1.16)	20	0.84	3.19 (1.35, 7.52)**	26	1.25	2.77 (1.24, 6.15)*
Prenatal care institution											
Medical center	33	4.69				4	1.11	3.60 (0.92, 14.1)			
Regional hospital	6	3.74				5	0.92	2.38 (0.14, 39.4)			
District hospital	5	2.60				5	0.66	2.64 (0.26, 26.4)			
Clinic	6	5.26				6	0.83	2.45 (0.16, 37.6)			
Comorbidity											
Diabetes	0	0.00	0	0.00	-	1	2.79	-	2	5.90	-
Hypertension	35	26.3	99	40.5	0.70 (0.47, 1.03)	20	6.23	3.11 (1.09, 8.89)*	25	8.22	2.90 (1.12, 7.53)*
Mild or unspecified pre-eclampsia	7	10.1				4	11.1	0.07 (0.01, 33.5)			
Severe pre-eclampsia	5	19.7				2	5.09	-			
Pre-eclampsia	3	100				2	13.2	-			
Eclampsia	1	12.6				0	0.00	-			
APS	1	1.54	6	4.94	0.62 (0.07, 6.01)	0	0.00	-	0	0.00	-
SS	0	0.00	1	4.96	-	0	0.00	-	0	0.00	-
Infection	31	9.39	88	11.9	0.89 (0.59, 1.35)	12	5.16	2.45 (0.80, 7.47)	10	5.61	2.67 (0.87, 8.15)
Stroke	3	8.77	10	11.0	0.65 (0.17, 2.59)	2	7.45	-	1	2.56	-
CKD	25	12.4	96	19.3	0.67 (0.43, 1.05)	5	138.3	0.50 (0.09, 2.82)	3	105.3	0.45 (0.09, 2.27)
Proteinuria	0	0.00	4	12.1	-	1	41.2	-	0	0.00	-
Immunosuppressant											
Prednisolone	50	4.35	159	7.55	0.83 (0.58, 1.18)	18	21.8	0.49 (0.21, 1.15)	19	16.6	0.55 (0.24, 1.26)
Endoxan	35	9.25	125	13.7	0.73 (0.50, 1.07)	0	0.00	-	3	15.9	0.61 (0.14, 2.64)
Imuran	36	5.65	124	8.79	0.76 (0.52, 1.10)	2	151.1	0.04 (0.01, 0.33)**	1	25.2	0.12 (0.01, 1.16)
Parity											
One	42	6.21				10	0.86	4.55 (1.67, 12.4)**			
Two	5	1.25				9	0.91	-			
More than three	3	3.18				1	0.44	-			

Rate^#^, incidence rate, per 1,000 person-years,

*p<0.05,

**p<0.01

^a^: multivariable analysis including geographic region, urbanization, occupation, hypertension, infection, stroke, CKD, proteinuria, prednisolone, Endoxan, and Imuran

^b^: multivariable analysis including geographic region, urbanization, prenatal care institution, hypertension, mild or unspecified pre-eclampsia, severe pre-eclampsia, pre-eclampsia, APS, SS, infection, stroke, CKD, proteinuria, prednisolone, Endoxan, Imuran, and parity

^c^: multivariable analysis including geographic region, urbanization, occupation, hypertension, APS, SS, infection, stroke, CKD, proteinuria, prednisolone, Endoxan, and Imuran

**Fig 2:**
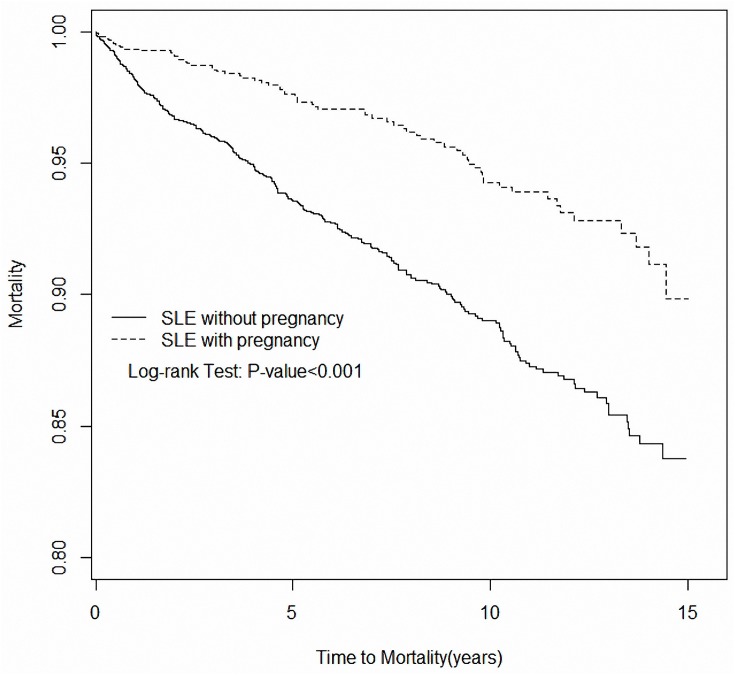
Survival curve for comparison between SLE patients with and without pregnancy.

The comparison of the overall survival in pregnant and nonpregnant SLE patients indicated that patient survival in the pregnant group was high with a statistical difference (*p* < 0.001) (Fig 2). After adjusting for other risk factors, a statistically improved survival was determined in pregnant SLE patients than those who were not pregnant (adjusted HR = 0.64, 95% CI = 0.48–0.87), particularly for SLE patients with a lower incidence of APS (adjusted HR = 0.04, 95% CI = 0.001–0.97) and infection (adjusted HR = 0.62, 95% CI = 0.41–0.92) (Table 3).

**Table 3:** Incidence densities and hazard ratio of overall mortality in pregnant and nonpregnant lupus patients.

	SLE				IRR* (95% CI)	Adjusted HR^†^ (95% CI)
	Pregnant		Non-pregnant			
	Case	Rate^#^	Case	Rate^#^		
Total	67	5.61	274	12.3	0.46 (0.35, 0.60)***	0.64 (0.48, 0.87)**
Comorbidity						
Diabetes	3	19.8	6	28.8	0.76 (0.19, 3.05)	0.51 (0.03, 8.47)
Hypertension	23	15.7	62	21.3	0.74 (0.46, 1.19)	0.89 (0.52, 1.53)
Mild or unspecified pre-eclampsia	8	11.1	0	0.00	-	-
Severe pre-eclampsia	7	25.4	0	0.00	-	-
Pre-eclampsia	1	22.9	0	0.00	-	-
Eclampsia	0	0.00	0	0.00	-	-
APS	1	1.53	17	13.9	0.11 (0.01, 0.80)	0.04 (0.001, 0.97)*
SS	0	0.0	1	4.89	-	-
Infection	38	11.0	170	21.9	0.50 (0.35, 0.72)***	0.62 (0.41, 0.92)*
Stroke	6	17.3	33	35.1	0.49 (0.21, 1.18)	0.66 (0.23, 1.91)
CKD	22	10.3	118	21.6	0.48 (0.30, 0.76)**	0.64 (0.39, 1.07)
Proteinuria	3	26.5	8	23.2	1.26 (0.33, 4.79)	7.23 (0.93, 56.3)
Immunosuppressant						
Prednisolone	66	5.62	263	12.0	0.47 (0.36, 0.61)***	0.67 (0.50, 0.91)*
Endoxan	42	10.6	165	17.0	0.62 (0.45, 0.88)**	0.74 (0.50, 1.09)
Imuran	50	7.66	186	12.7	0.60 (0.44, 0.82)**	0.77 (0.54, 1.10)
Parity						
One	50	7.18				
Two	14	3.48				
More than three	3	3.14				

Rate^#^, incidence rate, per 1,000 person-years

IRR*, incidence rate ratio

^†^: multivariable analysis including geographic region, urbanization, occupation, hypertension, APS, SS, infection, stroke, CKD, proteinuria, prednisolone, Endoxan, and Imuran.

*p<0.05,

**p<0.01,

***p<0.0001

## Discussion

The long-term outcome of ESRD in pregnant SLE patients was higher than that of non-SLE women who were pregnant or not. However, pregnant SLE patients showed better overall survival than nonpregnant SLE patients.

Previous studies generally considered renal impairment as a relatively benign condition in SLE patients. Patients with active LN in a few cases progressed to ESRD, which requires hemodialysis in the postpartum period [6, 17, 18]; however, data to investigate long-term renal outcomes in SLE patients after pregnancy are lacking. In a recent national study (the Nationwide Inpatient Sample dataset; NIS dataset), which investigated the risk of complications during pregnancy, approximately 20-fold higher risk of maternal mortality (OR: 17.8; 95% CI, 7.2–44) and a 3.7-fold higher risk (95% CI: 2.8–4.6) of renal failure after deliveries were determined in SLE pregnant patients compared with pregnant non-SLE women [19]. This finding suggested that the complex conditions, such as preeclampsia, eclampsia, and perinatal infection, are more likely to contribute to renal failure in SLE patients undergoing the gestational course. One case cohort (pregnant SLE patients) and three cohorts (nonpregnant SLE patients and non-SLE women who were pregnant and otherwise) were established in the present study to compare the incidences of ESRD after adjusting the described complications, including hypertension, diabetes, infection, APS, SS, pre-eclampsia, etc. Pregnant SLE patients exhibited an increased risk of ESRD (HR = 3.19, 95% CI, 1.35–7.52), which was consistent with the findings of the previous study [19] and indicated that the risk of ESRD should not be disregarded in SLE patients after pregnancy.

Risk factors related to ESRD in SLE patients after pregnancy were identified in the current study. Hypertension was associated with the risk of ESRD in pregnant SLE patients. The role of hypertension as a contributor to ESRD in most pregnant women was previously clarified. A study that evaluates the effect of hypertension on renal outcomes in women after pregnancy determined that hypertensive women showed a 2.72-fold increase in the risk of ESRD after pregnancy compared with those without hypertension [20]. The significantly higher risk of hypertensive disorders, including hypertension and preeclampsia, in SLE pregnant patients was considered to be highly associated with the flare-up of LN, and has been suggested to lead to cardiovascular diseases in the future [4, 21]. In our analysis, hypertension was associated with increased the risk of ESRD, which was consistent with the results in a previous study [19].

The current analysis determined that pregnant SLE patients showed a slightly lower incidence of ESRD than nonpregnant SLE patients and no significantly increased risk of ESRD after adjusting for potential factors. The findings suggested that the influence of pregnancy on adverse outcomes in SLE patients appear to be less than expected. Some potential explanations may account for these findings. The mean period of SLE in pregnant SLE patients identified to pregnancy was 4.93± 3.46 years, which was longer than the recommended period of six months. These patients showed significantly lower prevalence rates for the baseline comorbidities compared with nonpregnant SLE patients. Interpreting the SLE status during conception is difficult because of the lack of immunological and biochemical data in the NHIRD database. However, approximately 80% of all SLE patients in the current study received the treatment and multidisciplinary care consisting of various specialists, including rheumatologists, nephrologists, and obstetric doctors, in referral medical centers. These patients ordinarily decided to continue their pregnancy based on their relatively stable clinical conditions. Pregnant SLE patients should have a relatively low immunological risk. To a certain extent, the findings may suggest the safety and possibility of conception in SLE patients if these patients fully comply with the current recommendation [22]. This practice may reduce complications caused by disease activity and the side effects of intensive immunosuppressive therapy, thereby reducing the risk of infection [4]. Patients with severe SLE generally take intensive immunosuppressant, such as Endoxan. Approximately 98% of SLE patients received the prednisolone immunosuppressant, 30%–40% received Endoxan, and 55%–65% received Imuran. Despite adjusting for these immunosuppressants, pregnant SLE patients exhibited better survival than those who were not pregnant. SLE patients with serious clinical conditions might be advised to avoid pregnancy, which may partially explain the lower mortality in pregnant SLE patients than those who were not pregnant. Therefore, pregnant SLE patients in the current study showed better baseline conditions than those who were not pregnant. The findings indicated the problem of high self-selectivity in the case cohort. The probability of extrapolation may be reduced. The results emphasized the importance of a multispecialty approach in managing SLE patients. Monitoring disease activity and the early recognition of hypertensive disorders are crucial to attenuate the harmful effects on the maternal kidneys. A major limitation in the study is the lack of clinical data, including the titers of disease activity, urine protein, blood pressure record, and serum creatinine. CKD was used to identify patients with poor serum creatinine, and the full-model analysis was adjusted further. The details of the subclass of LN, which is critical to kidney outcomes, cannot to be analyzed further in this study. SLE is a catastrophic illness. Thus, the end date of the coverage was considered as the date of death. The overall mortalities between pregnant and nonpregnant SLE patients could be precisely evaluated in the present analysis in comparison with non-SLE patients. Some of the comorbidities ICD9 coding would be underestimated, such as proteinuria and antiphospholipid syndrome. This condition could result in the non-differential misclassification, and the results would be underestimated. Other laboratory studies are necessary to further elucidate the roles of these comorbidities on the relationships among SLE, pregnancy, and ESRD incidence or overall mortality.

## Conclusion

The long-term outcome of ESRD in pregnant SLE patients was higher than that of non-SLE women who were pregnant or not. In addition, pregnant SLE patients showed better overall survival than SLE patients who are not pregnant. Multicenter recruitment and clinical information collection merit further exploration of the combination of SLE and pregnancy for ESRD (or mortality) incidence in future studies.
